# Economic crises, behavioral changes and hospitalization due to affective disorders in Brazil between 2003 and 2017: a nationwide cross-sectional study

**DOI:** 10.1590/1516-3180.2019.0127.R2.19112019

**Published:** 2020-04-09

**Authors:** André Oliveira Werneck, Rômulo Araújo Fernandes, Danilo Rodrigues Silva

**Affiliations:** I MSc. Researcher, Grupo de Investigações Científicas Relacionados à Atividade Física (GICRAF), Laboratório de Investigação em Exercício (LIVE), Department of Physical Education, Universidade Estadual Paulista (UNESP), Presidente Prudente (SP), Brazil.; II PhD. Associate Professor, Grupo de Investigações Científicas Relacionados à Atividade Física (GICRAF), Laboratório de Investigação em Exercício (LIVE), Department of Physical Education, Universidade Estadual Paulista (UNESP), Presidente Prudente (SP), Brazil.; III PhD. Associate Professor, Department of Physical Education, Universidade Federal de Sergipe (UFS), São Cristóvão (SE), Brazil.

**Keywords:** Economic recession, Depression, Exercise, Economic crises, Physical activity, Lifestyle behavior

## Abstract

Our aim was to analyze hospitalization due to affective disorders in Brazil from 2003 to 2017 and the possible association with economic indicators during crises. We used data on hospitalizations due to affective disorders within the Brazilian National Health System, obtained from DATASUS; data on health-related behavior (television-viewing and physical activity) from the VIGITEL database; and economic data from the World Bank database. We found that the numbers of hospitalizations increased one year after the 2009 crisis and one year after the 2016 crisis. Negative changes in health-related behavior also followed changes in the numbers of hospitalizations due to affective disorders.

## INTRODUCTION

After the years of economic prosperity in Brazil in the early 2000s, with the success of the “real plan” and the reductions in inflation and unemployment and increases in healthcare investments,^1^^,^^2^ Brazil experienced two economic crises. The first was the world crisis of 2008-2009 which, although not large in Brazil, was responsible for a 0.1% deflation in the gross domestic product (GDP) in 2009. This was the first deflation in Brazil since 1993.^1^ After this, Brazil returned to a period of growth in GDP, with social, educational and public health achievements up to 2015, when the greatest recession in Brazilian history began. The 2016 crisis led to successive years with GDP deflation of more than 3%,^1^ along with an important political crisis that included a presidential impeachment.

During these economic and political crises, the percentage of GDP expended on the healthcare system started to reduce, through the impact of several austerity policies.^2^ With the economic crises, the levels of unemployment increased. Higher unemployment has the consequences of affecting economic power, social security and job stability (through labor-law deregulation). This may have substantial associations with mental health.^3^^,^^4^

## OBJECTIVE

Our aim was to analyze occurrences of hospitalizations due to affective disorders in Brazil between 2003 and 2017, along with the economic oscillations and the prevalences of different types of health-related behavior.

## METHODS

This ecological study used data on hospitalizations due to affective disorders (International Classification of Diseases 10^th^ edition, ICD 10; codes F30-F39). These data were collected from the morbidity and mortality surveillance system (DATASUS), which records all hospitalizations within the Brazilian National Health System (Sistema Único de Saúde, SUS). DATASUS has national coverage, with registration of approximately 11 million hospital admissions per year.^5^

Brazilian data on GDP and unemployment were collected from the World Bank database covering the years 2003-2017.^1^ Data on health-related behavior (television-viewing and physical activity) were obtained from the telephone survey-based surveillance system for risk and protective factors for chronic diseases (VIGITEL). VIGITEL is a survey that has been conducted annually since 2006 using a probabilistic sample of adults (≥ 18 years) in 26 Brazilian state capitals and the Federal District. Here, we used data from 2006 to 2016. More details on VIGITEL are available elsewhere.^6^ Data on hospitalizations, economic oscillations and behaviors were cross-referenced to analyze possible interrelationships.

Our statistical approach was to provide crude values for the numbers of hospitalizations due to affective disorders, prevalence of physical activity, prevalence of elevated time spent sitting down, GDP and unemployment rate, in order to obtain an ecological perspective. Moreover, we created an indicator for hospitalization ratio, which comprised the number of hospitalizations due to affective disorders per 1000 all-cause hospitalizations.

## RESULTS

**Figure 1.**
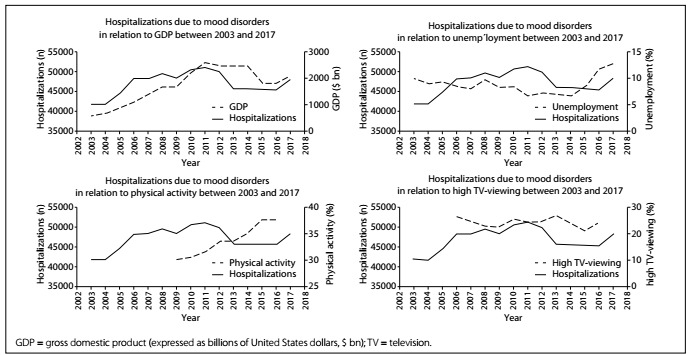
Association of economic and behavioral factors with hospitalizations due to mood disorders between 2003 and 2017.

Descriptive statistics regarding the prevalence of hospitalizations due to affective disorders, economic oscillations and health-risk behaviors are presented in Figure 1 and Table 1. Greater numbers of hospitalizations due to affective disorders were found during 2010 and 2011, one year after the economic recession of 2009-2010. Moreover, after the economic recession of 2016, the number of hospitalizations due to affective disorders increased again in 2017. Moreover, when the number of hospitalizations due to affective disorders increased in 2017, the prevalence of high levels of television-viewing also increased, while the levels of physical activity practice remained stagnated. This contrasted with the increase that had been observed over the period between 2006 and 2016.

**Table 1.** Frequency of hospitalizations due to mood disorders in relation to economic and behavioral factors

Year	Hospitalizations (n)	Hospitalization ratio (hospitalizations / 1000 all-cause hospitalizations)	GDP ($ bn)	Unemployment (%)	PA (%)	High TV-viewing (%)
2003	41,870	0.36	558.31	9.73	-	-
2004	41,814	3.64	669.32	8.89	-	-
2005	44,509	3.89	891.63	9.31	-	-
2006	48,211	4.25	1,107.64	8.39	-	28.0
2007	48,297	4.26	1,397.08	8.09	-	26.3
2008	49,469	4.60	1,695.82	9.46	-	24.6
2009	48,406	4.35	1,667.02	8.28	30.3	24.0
2010	50,578	4.45	2,208.87	8.36	30.5	27.3
2011	51,216	4.54	2,616.20	6.69	31.6	25.9
2012	49,846	4.49	2,465.19	7.19	33.5	26.4
2013	45,779	4.09	2,472.81	6.99	33.8	28.6
2014	45,879	4.05	2,455.99	6.67	35.3	25.3
2015	45,618	4.01	1,802.21	8.44	37.6	22.5
2016	45,419	4.02	1,793.99	11.61	37.6	25.7
2017	48,202	4.20	2,055.51	12.88	-	-

Note: Hospitalizations refers to hospitalizations due to mood disorders. Hospitalization ratio: hospitalizations due to affective disorders per 1000 all-cause hospitalizations.

GDP = gross domestic product (expressed as billions of United States dollars, $ bn); PA = physical activity; TV = television.

## DISCUSSION

We observed that periods after recessions (with a reduction in GDP and an increase in unemployment) were characterized by an increase in hospitalizations due to mood disorders, especially during 2010 and 2017. Regarding important mental health correlates,^7^ television-viewing increased especially during the recession period and followed the kinetics of hospitalizations due to mood disorders. In addition, when the physical activity level of the population stopped increasing, the number of hospitalizations due to mood disorders increased (between 2016 and 2017).

Worldwide, evidence of several negative changes to health outcomes during crisis periods has been found, especially when these have been followed by austerity policies within healthcare systems. Studies on the most recent crisis in European countries found that increases in communicable and non-communicable diseases occurred.^3^ One of the main examples comes from Greece, which suffered severe austerity policies in 2010. This was the country that suffered the greatest number of public health consequences.^4^

Although the greatest Brazilian crisis is still recent, the negative impact on public health can already be observed. In times with increased unemployment, labor-law deregulation and social insecurity, mental health seems be affected in different ways.^3^^,^^4^ One possible explanation for our findings could be the migration of people from private healthcare to the public healthcare system.^2^ However, we found similar results when taking into consideration the hospitalization ratio.

Specifically, regarding the impact of economic crises on mental health, previous studies found that economic crises were associated with increases in the prevalences of several negative mental health outcomes, especially stress, anxiety and depressive symptoms. These were correlated with recent economic crises in Italy and Spain.^8^^,^^9^

Moreover, employment status seems to be an important mediator of the negative impact of economic crises on mental health. Going beyond unemployment itself, a previous study investigating the impact of an economic crisis in the Netherlands found that working conditions became worse during the crisis and that work insecurity increased. There was also an association with negative health outcomes.^10^

Economic crises can also affect lifestyle behaviors such as physical activity levels, television-viewing and diet.^11^^,^^12^ Consequently, adoption of unhealthy lifestyles can also be correlated with negative mental health outcomes.^7^^,^^13^^,^^14^

Given the ecological approach adopted, we do not intend to establish causality between economic crises, health-related behaviors and hospitalizations due to affective disorders. Naturally, many factors could explain changes in the parameters analyzed.

Nevertheless, these data show that a tendency towards changes in the same direction occurs, which may indicate co-occurrence. These findings at least provide empirical evidence regarding the changes at population level that follow periods of economic recess. They also highlight the need for further studies to clarify these interrelationships.

## CONCLUSION

These findings confirm the potentially harmful effects of an economic crisis on public health. Similar to the experience of other countries, Brazil will probably feel the negative impacts of the last recession period for some time yet and will take time to recover. Most importantly, this is a period for reflection and decision-making. Brazil could continue to follow examples of strong austerity, with freezing of social spending, as was done in Greece, Spain and Portugal in response to the European crisis; or it could adopt examples such as that of Iceland, which faced up to the financial recession through implementing sustainable measures focusing on social protection.

## References

[1] World Bank (2018). World Development Indicators.

[2] Massuda A, Hone T, Leles FAG, de Castro MC, Atun R. (2018). The Brazilian health system at crossroads: progress, crisis and resilience. BMJ Glob Health.

[3] Stuckler D, Reeves A, Loopstra R, Karanikolos M, McKee M. (2017). Austerity and health: The impact in the UK and Europe. Eur J Public Health.

[4] Global Burden of Disease 2016 Greece Collaborators (2018). The burden of disease in Greece, health loss, risk factors, and health financing, 2000-16: an analysis of the Global Burden of Disease Study 2016. Lancet Public Health.

[5] Paim J, Travassos C, Almeida C, Bahia L, Macinko J. (2011). The Brazilian health system: history, advances, and challenges. Lancet.

[6] Ministério da Saúde (BR). Secretaria de Vigilância em Saúde (2019). Vigitel Brasil 2019: Vigilância de Fatores de Risco e Proteção Para Doenças Crônicas Por Inquérito Telefônico.

[7] Vancampfort D, Firth J, Schuch FB (2017). Sedentary behavior and physical activity levels in people with schizophrenia, bipolar disorder and major depressive disorder: a global systematic review and meta-analysis. World Psychiatry.

[8] Odone A, Landriscina T, Amerio A, Costa G. (2018). The impact of the current economic crisis on mental health in Italy: evidence from two representative national surveys. Eur J Public Health.

[9] Salvador-Carulla L, Roca M. (2013). Mental health impact of the economic crisis in Spain. Int Psychiatry.

[10] ten Have M, van Dorsselaer S, de Graaf R. (2015). The association between type and number of adverse working conditions and mental health during a time of economic crisis (2010-2012). Soc Psychiatry Psychiatr Epidemiol.

[11] Foscolou A, Tyrovolas S, Soulis G (2017). The Impact of the Financial Crisis on Lifestyle Health Determinants Among Older Adults Living in the Mediterranean Region: The Multinational MEDIS Study (2005-2015). J Prev Med Public Health.

[12] Scuri S, Tesauro M, Petrelli F (2018). Implications of modified food choices and food-related lifestyles following the economic crisis in the Marche Region of Italy. Ann Ig.

[13] Firth J, Stubbs B, Teasdale SB (2018). Diet as a hot topic in psychiatry: a population-scale study of nutritional intake and inflammatory potential in severe mental illness. World Psychiatry.

[14] Schuch FB, Vancampfort D, Firth J (2018). Physical Activity and Incident Depression: A Meta-Analysis of Prospective Cohort Studies. Am J Psychiatry.

